# Needles in the EST Haystack: Large-Scale Identification and Analysis of Excretory-Secretory (ES) Proteins in Parasitic Nematodes Using Expressed Sequence Tags (ESTs)

**DOI:** 10.1371/journal.pntd.0000301

**Published:** 2008-09-24

**Authors:** Shivashankar H. Nagaraj, Robin B. Gasser, Shoba Ranganathan

**Affiliations:** 1 Department of Chemistry and Biomolecular Sciences, Macquarie University, Sydney, New South Wales, Australia; 2 Department of Veterinary Science, The University of Melbourne, Werribee, Victoria, Australia; 3 Department of Biochemistry, Yong Loo Lin School of Medicine, National University of Singapore, Singapore; Yale Child Health Research Center, United States of America

## Abstract

**Background:**

Parasitic nematodes of humans, other animals and plants continue to impose a significant public health and economic burden worldwide, due to the diseases they cause. Promising antiparasitic drug and vaccine candidates have been discovered from excreted or secreted (ES) proteins released from the parasite and exposed to the immune system of the host. Mining the entire expressed sequence tag (EST) data available from parasitic nematodes represents an approach to discover such ES targets.

**Methods and Findings:**

In this study, we predicted, using EST2Secretome, a novel, high-throughput, computational workflow system, 4,710 ES proteins from 452,134 ESTs derived from 39 different species of nematodes, parasitic in animals (including humans) or plants. In total, 2,632, 786, and 1,292 ES proteins were predicted for animal-, human-, and plant-parasitic nematodes. Subsequently, we systematically analysed ES proteins using computational methods. Of these 4,710 proteins, 2,490 (52.8%) had orthologues in *Caenorhabditis elegans*, whereas 621 (13.8%) appeared to be novel, currently having no significant match to any molecule available in public databases. Of the *C. elegans* homologues, 267 had strong “loss-of-function” phenotypes by RNA interference (RNAi) in this nematode. We could functionally classify 1,948 (41.3%) sequences using the Gene Ontology (GO) terms, establish pathway associations for 573 (12.2%) sequences using Kyoto Encyclopaedia of Genes and Genomes (KEGG), and identify protein interaction partners for 1,774 (37.6%) molecules. We also mapped 758 (16.1%) proteins to protein domains including the nematode-specific protein family “transthyretin-like” and “chromadorea ALT,” considered as vaccine candidates against filariasis in humans.

**Conclusions:**

We report the large-scale analysis of ES proteins inferred from EST data for a range of parasitic nematodes. This set of ES proteins provides an inventory of known and novel members of ES proteins as a foundation for studies focused on understanding the biology of parasitic nematodes and their interactions with their hosts, as well as for the development of novel drugs or vaccines for parasite intervention and control.

## Author Summary

Excretory-secretory (ES) proteins are an important class of proteins in many organisms, spanning from bacteria to human beings, and are potential drug targets for several diseases. In this study, we first developed a software platform, EST2Secretome, comprised of carefully selected computational tools to identify and analyse ES proteins from expressed sequence tags (ESTs). By employing EST2Secretome, we analysed 4,710 ES proteins derived from 0.5 million ESTs for 39 economically important and disease-causing parasites from the phylum Nematoda. Several known and novel ES proteins that were either parasite- or nematode-specific were discovered, focussing on those that are either absent from or very divergent from similar molecules in their animal or plant hosts. In addition, we found many nematode-specific protein families of domains “transthyretin-like” and “chromadorea ALT,” considered vaccine candidates for filariasis in humans. We report numerous *C. elegans* homologues with loss-of-function RNAi phenotypes essential for parasite survival and therefore potential targets for parasite intervention. Overall, by developing freely available software to analyse large-scale EST data, we enabled researchers working on parasites for neglected tropical diseases to select specific genes and/or proteins to carry out directed functional assays for demystifying the molecular complexities of host–parasite interactions in a cell.

## Introduction

Molecules secreted by a cell, often referred to excretory/secretory (ES) products, play pivotal biological roles across a diverse range of taxa, ranging from bacteria to mammals [1]. ES proteins can represent 8±20% of the proteome of an organism [1],[2]. ES proteins include functionally diverse classes of molecules, such as cytokines, chemokines, hormones, digestive enzymes, antibodies, extracellular proteinases, morphogens, toxins and antimicrobial peptides. Some of these proteins are known to be involved in vital biological processes, including cell adhesion, cell migration, cell-cell communication, differentiation, proliferation, morphogenesis and the regulation of immune responses [3]. ES proteins can circulate throughout the body of an organism (in the extracellular space), are localized to or released from the cell surface, making them readily accessible to drugs and/or the immune system. These characteristics make them attractive as targets for novel therapeutics, which are currently the focus of major drug discovery research programmes [4]. For example, knowledge of the molecular basis of secretory pathways in bacteria has facilitated the rational design of heterologous protein production pathways in biotechnology and in the development of novel antibiotics. From a more fundamental perspective, proteins secreted by pathogens are of particular interest in relation to the pathogen-host interactions, because they are present or active at the interface between the parasite and host cells, and can regulate the host response and/or cause disease [5],[6].

ES proteins have long been the focus of biochemical and immunological studies of parasitic helminths, as such worms secrete biologically active mediators which can modify or customize their niche within the host, in order to evade immune attack or to regulate or stimulate a particular host response [7],[8],[9],[10]. Parasitic nematodes are responsible for a range of neglected tropical diseases, such as ancylostomatosis, necatoriasis, lymphatic filariasis, onchocerciasis, ascariasis and strongyloidiasis in humans [11],[12], and others can cause massive production or economic losses to farmers as well as to animal and plant industries [13].

There have been efforts to identify and characterize ES proteins in different parasitic nematodes in various studies. For instance, Robinson et al. [14] used a proteomic approach to identify ES glycoproteins in *Trichinella spiralis*, an enoplid nematode (or trichina) of musculature. In another effort, Yatsuda et al. [9] undertook an analysis of ES products from *Haemonchus contortus* (barber's pole worm), a parasite of small ruminants; these authors identified several novel and known proteins but were only able (based on comparative analysis) to investigate known proteins, such as serine, metallo- and aspartyl- proteases and the microsomal peptidase H11, a vaccine candidate, previously recognised as a “hidden antigen” [15]. The precise role of ES proteins from parasitic nematodes in mediating cellular processes is largely unknown due to the difficulty in experimentally assigning function to individual proteins [14]. In this context, computational approaches applied to identify and annotate ES proteins have significantly complemented experimental studies of different cells, tissues, organs and organisms. For example, in an early study, Grimmond et al. [16] developed a computational strategy to identify and functionally classify secreted proteins in the mouse, based on the presence of a cleavable signal peptide (required for its entry into the secretory pathway), along with the lack of any transmembrane (TM) domain or intracellular localization signals, in full-length molecules. This study was followed by the computational reconstruction of the secretome in human skeletal muscle from protein sequence data by Bortoluzzi et al. [17]. Also, Martinez et al. [18] identified and annotated the secreted proteins involved in the early development of the kidney in the mouse from microarray ‘expression’ profiling, using computational strategies.

While expressed sequence tag (EST) data have been mined for many interesting functional molecules [19],[20], predicting ES proteins from ESTs has been relatively uncommon. For example, Vanholme et al. [21] identified putative secreted proteins from EST data sets for the plant parasitic nematode, *Heterodera schachtii*. Harcus et al. [22] investigated the signal sequences inferred from the EST data for the parasitic nematode *Nippostrongylus brasiliensis*, and related them to “accelerated evolution” of secreted proteins in this parasite, compared with host or non-parasitic organisms. Ranganathan et al. [23] identified ES proteins from EST data for the bovine lungworm, *Dictyocaulus viviparus*, whereas Nagaraj et al. [24] identified and classified putative secreted proteins from *Trichostrongylus vitrinus*, a parasitic nematode of ruminants and suggested some molecules as candidates for developing novel anthelmintics or vaccines. One of the suggested molecules, *Tv-stp1*, was investigated further and functionality established [25].

While single EST or protein data sets have been examined for the presence of secretory or ES proteins, large-scale analysis has not been conducted to date, due to the lack of effective high-throughput, computational pipelines for analysis [16]. Recently, we designed a high-throughput EST analysis pipeline, ESTExplorer [26] to provide comprehensive DNA and protein-level annotations. Based on earlier work [23],[24], ESTExplorer has been adapted to predict ES proteins with high confidence, and then provide extensive annotation, including Gene Ontologies (GO), pathway mapping, protein domain identification and predict protein-protein interactions. Our new pipeline, EST2Secretome, is a freely available web server that can directly process vast amounts of EST data or entire proteomes.

In the present study, approximately 500,000 ESTs, representing 39 economically important and disease-causing parasitic nematodes of humans, other animals and plants, were subjected to a comprehensive analysis and detailed annotation of inferred ES proteins using EST2Secretome, with specific reference to candidate molecules already being assessed as intervention targets. We compared the predicted ES proteins with those inferred from the free-living nematode *C. elegans,* to establish whether these proteins could be nematode-specific and propose their functionality. Also, we examined whether the ES proteins had homologues in their respective hosts (animal, human or plant), as such proteins and their genes are less likely to be useful as intervention targets. Pathway, interactome and literature-based ES protein analyses have assisted in gleaning sets of candidate molecules for future experimental studies. The present results lay a foundation for understanding the functional complexity of ES proteins from parasitic nematodes and their interactions with other proteins (within the nematodes) and/or with host proteomes.

## Materials and Methods

### Description of EST2Secretome

EST2Secretome (http://EST2secretome.biolinfo.org/) is a comprehensive workflow system comprising carefully selected computational tools to identify and annotate ES proteins inferred from ESTs. EST2Secretome provides a user-friendly interface and detailed online help to assist researchers in the analysis of EST data sets for ES proteins. The workflow can be divided into three phases, with Phase I dedicated to pre-processing, assembly and conceptual translation, similar to that of ESTExplorer (details described in Nagaraj et al. [26]). In Phase II, putative ES proteins are identified based on the presence of signal sequences and the absence of transmembrane helices. Phase III contains a comprehensive annotation layer, comprising a suite of bioinformatic tools to annotate the ES proteins inferred in Phase II. ESTs can be submitted to Phase I for EST pre-processing, assembly and conceptual translation, followed by the identification of putative ES proteins in Phase II and annotation in Phase III. Alternatively, instead of EST data, protein sequences may be submitted directly to Phase II to identify putative ES proteins and functionally annotate them in Phase III.

**Figure 1:**
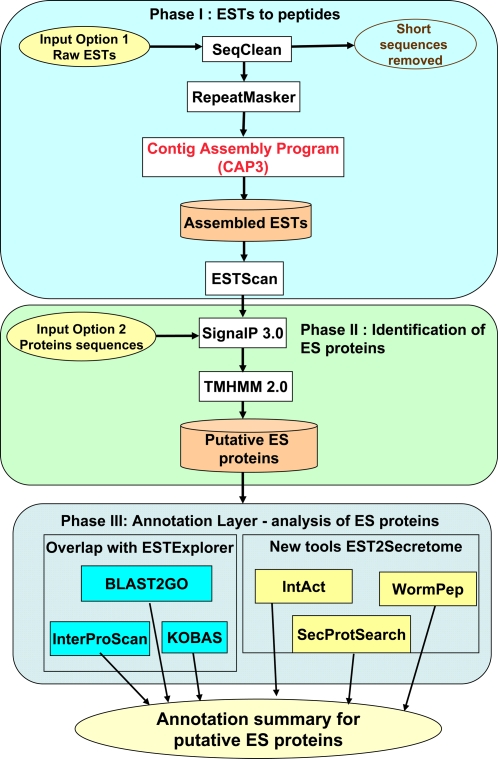
Schematic representation of EST2Secretome workflow. EST2Secretome analysis comprising Phase I: pre-processing, assembly and conceptual translation, Phase II: identification of putative excretory-secretory (ES) proteins and Phase III: annotation of ES proteins using a suite of computational tools.

Phase I of EST2Secretome shares SeqClean, RepeatMasker and CAP3 (contig assembly program) programs with ESTExplorer [26], based on the analysis presented elsewhere [20]. The contig and singleton sequences generated by CAP3 are transferred to the program ESTScan [27] for conceptual translation into proteins, using the genetic code from the nearest organism. EST2Secretome currently implements the genetic codes for 15 organisms, covering the most studied organisms, including human, mouse, rat, pig, dog, chicken, rice, wheat, thale cress (*Arabidopsis thaliana*), zebrafish, fly, yeast and a free-living roundworm (*Caenorhabditis elegans*) (Figure 1).

In Phase II, putative ES proteins are identified from the protein sequences generated in Phase I, using the two programs SignalP [28] and TMHMM [29] (Figure 1). SignalP first checks whether a signal sequence [30] is predicted both the artificial neural network and the hidden Markov model probability scores (SignalPNN and SignalP-HMM), using default parameters that can be modified by experienced users. Subsequently, all proteins with signal sequences are passed on to TMHMM [29], a hidden Markov model-based transmembrane helix prediction program, to “filter out” of transmembrane proteins. The subset lacking transmembrane helices is selected as ES proteins for further annotation.

Phase III is the annotation layer, comprising a suite of six computational tools for the functional annotation of ES proteins, of which the first three (Gene Ontology using BLAST2GO, InterProScan and pathway mapping using KOBAS) are also implemented in ESTExplorer and described elsewhere [26]. The other three components are unique to EST2Secretome and incorporate protein BLAST searches against three different data sets derived from Wormpep [31] for locating nematode homologues, IntAct [32] for protein-protein interaction data and a non-redundant known secreted protein database (SecProtSearch) derived from the literature, the secreted protein database, SPD [33] and the manually curated signal peptide database, SPdb [34]. Mapping to Wormpep gives a list of homologous proteins in *C. elegans*, linked to WormBase [31]. Homologues from the IntAct database are determined using the concept of interlogs (evolutionarily conserved interactions identified by conservation among homologous proteins in different species) and are linked to all molecular interaction partners of homologous proteins. EST2Secretome provides a link to the relevant interlog page at IntAct, containing all interaction partners. The interaction data culled from these interlogs can be extrapolated to predict protein interactions of the query sequence, for validation by complementary double-stranded RNA interference (RNAi), gene deletion or fluorescence-based interaction studies. The final module compares the query sequence to a specialised data set of known secreted proteins (SecProtSearch), in order to identify orthologous secreted proteins, which would provide a second level of validation for the ES protein dataset. Phase III (Figure 1) thus allows extensive characterization and validation of ES proteins predicted by EST2Secretome.

Once an EST (or a protein dataset) has been submitted to EST2Secretome, a status page is accessible, for the monitoring of the progress of the analysis, at the program level. As each selected program is completed, the status page is updated and the output from that program becomes available. The outcome from each run is summarized, with links to output files from each selected program being listed. When a large dataset is analysed using a workflow, it is challenging to collate the results of the analysis from multiple steps. To address this issue, EST2Secretome provides a summary file for each ES protein, comprising the assembled contig/singleton sequence, the peptide sequence and all the annotations (such as homologous proteins, protein domains, pathways and interaction partners).

### Implementation of EST2Secretome

The details of the EST2Secretome workflow, including the software and hardware used, are provided on the website. A detailed tutorial, frequently asked questions (FAQ) and sample EST and protein datasets are available online for the effective use of EST2Secretome.

### Identification and analysis of ES proteins

452,134 ESTs (as at 18 December 2007) from 39 parasitic nematodes (7 from human, 18 from other animals and 14 from plants, Table 1) were downloaded from dbEST [19]. ESTs from each organism were submitted to Phase I of EST2Secretome, where they were pre-processed (SeqClean and RepeatMasker), aligned/clustered using CAP3 [35], with a minimum sequence overlap length “cut-off” of 30 bases and an identity threshold of 90%, for the removal of flanking vector and adapter sequences, followed by assembly. These high quality contigs and singletons were conceptually translated using ESTScan [27], based on a “*smat*” matrix, generated from available mRNA data for each organism. When the *smat* file for a specific organism is not available, the nearest well-studied organism has to be selected as a reference, based on taxonomy, and its *smat* file is used instead. We used data (25,481 cDNA sequences) from *C. elegans* (as it is the best studied nematode) for the generation of the *smat* file. The conceptually translated peptide data were transferred to Phase II of EST2Secretome, for the prediction of ES proteins, by sequentially running the SignalP [28] and TMHMM [29] programs. For SignalP, the threshold values for the D-score and the Signal peptide probability were both set to 0.5, based on a validation carried out for 1946 sequences of experimentally verified signal peptides from the recently updated SPdb [34], with an accuracy of prediction of 98.1%. Any protein that simultaneously fulfilled the threshold set for both the D-score and the Signal peptide probability score, was classified as a secretory-excretory (ES) protein. Inferred ES proteins were then tested for the presence of transmembrane domains using the transmembrane helix and membrane topology prediction program, TMHMM [29] and sequences containing predicted transmembrane regions were eliminated to yield only those proteins that were predicted as destined for secretion.

**Table 1:** Nematodes parasitic in humans, other animals and plants (listed alphabetically for each host group) and their principal (definitive) hosts, selected for the analysis of excretory-secretory (ES) proteins inferred from EST data available from current databases (including dbEST).

Number	Nematode Parasite	Common name or description	Principal [definitive] host or host group	Disease or common name of disaease	Number of ESTs analysed
**Animal host**					
1	*Ancylostoma ceylanicum*	Hookworm	Human, dog and cat	Hookworm disease	10712
2	*Ascaris lumbricoides*	Common roundworm	Human	Ascariasis	1863
3	*Brugia malayi*	Filarial worm	Human	Lymphatic filariasis (elephantiasis)	26215
4	*Necator americanus*	Hookworm	Human	Hookworm disease	5032
5	*Onchocerca volvulus*	-	Human	Onchocerciasis (river blindness)	14974
6	*Strongyloides stercoralis*	-	Human	Strongyloidiasis	11392
7	*Wuchereria bancrofti*	Filarial worm	Human	Lymphatic filariasis (elephantiasis)	4847
1	*Ancylostoma caninum*	Canine hookworm	Dog	Hookworm disease	80551
2	*Ascaris suum*	Large common roundworm of pigs	Pig	Ascariasis	40771
3	*Dirofilaria immitis*	Canine heartworm	Dog	Heartworm disease	4005
4	*Dictyocaulus viviparus*	Bovine lungworm	Cattle	Dictyocaulosis, parasitic bronchitis, husk	4469
5	*Haemonchus contortus*	Barber's pole worm	Small ruminant (sheep and goat)	Haemonchosis	21975
6	*Litomosoides sigmodontis*	Filarial worm	Rodent	Rodent filariasis	2699
7	*Nippostrongylus brasiliensis*	Intestinal parasite of rats	Rodent	Nippostrongylosis	8238
8	*Ostertagia ostertagi*	Brown stomach worm of cattle	Cattle (and other bovids)	Ostertagiasis	7006
9	*Oesophagostomum dentatum*	Nodule worm of pigs	Pig	Oesophagostomiasis	328
10	*Onchocerca ochengi*	-	Cattle	Onchocerciasis	60
11	*Parastrongyloides trichosuri*	Intestinal parasite of Australian brush-tail(ed) possum	Possum	-	7963
12	*Strongyloides ratti*	-	Rat	Strongyloidiasis	14761
13	*Teladorsagia circumcincta*	Brown stomach worm	Small ruminant (sheep and goat)	Ostertagiasis	6058
14	*Toxocara canis*	Common roundworm of dogs	Dog	Toxocariasis (larval toxocariasis in intermediate host)	4889
15	*Trichinella spiralis*	Trichina	Mammals, including rats, pigs, canids, humans	Trichinellosis	21985
16	*Trichuris muris*	Murine whipworm	Rodent	Intestinal diseases	2714
17	*Trichuris vulpis*	Canine whipworm	Canids	Trichuriasis	3063
18	*Trichostrongylus vitrinus*	Black scour worm	Sheep and goat	Trichostrongylosis	1000
**Plant host**					
1	*Globodera pallida*	White cyst nematode, Potato cyst nematode	Tomato, eggplant	Potato cyst disease	4378
2	*Globodera rostochiensis*	Potato cyst nematode	Potato, tomato, eggplant	Potato cyst disease, yellowing or wilting of foliage	11851
3	*Heterodera glycines*	Soybean cyst nematode	Soybean	Soybean cyst disease, yellow dwarf	24444
4	*Heterodera schachtii*	Sugar beat cyst nematode	Sugar beet, cabbage, cauliflower, brussel sprouts, mustard, radish, spinach, chard	Sugar beat cyst disease, stunted, wilt	2818
5	*Meloidogyne arenaria*	Peanut root-knot nematode	Peanut, vegetables, grasses, fruit, ornamentals and tobacco	Root-knot disease, large galls on roots, pegs, pods and runners	5018
6	*Meloidogyne chitwoodi*	Columbia root-knot nematode	Various plants, including potato, barley, wheat and alfalfa	Root-knot disease, nematode-induced blemish in tubers, stunting	12218
7	*Meloidogyne hapla*	Northern root-knot nematode	>500 plant hosts, including vegetables, clover, alfalfa and ornamentals	root-knot disease, galls on roots; poor growth; shortened lifespan of the vine	24452
8	*Meloidogyne incognita*	Cotton Root-knot Nematode	Cotton, tobacco, peanut and fibre crops	Root-knot disease; causes wilting of the infected plant that leads to death, Blackshank of tobacco	20334
9	*Meloidogyne javanica*	Root Knot Nematode	>770 species of plants, including peanut. sugarcane and fibre crops	Root-knot disease	7587
10	*Meloidogyne paranaensis*	Coffee root-knot nematode	Coffee	Coffee root-knot disease resulting in decline, dieback of coffee trees	3710
11	*Pratylenchus penetrans*	lesion nematode; meadow nematode	Apple, cherry, peach	Necrotic lesions and chlorosis	1928
12	*Pratylenchus vulnus*	Walnut meadow nematode or Walnut root-lesion nematode	Apple, cherry, peach	Invades the cortex of roots, tubers, and results in necrotic lesions	5812
13	*Radopholus similis*	Burrowing nematode	Banana, citrus	Migratory endoparasite; causes spreading decline	7380
14	*Xiphinema index*	Dagger nematode	Grape	Root stunting and tip galling	9351

### Annotation of ES proteins

Inferred ES proteins were annotated by selecting all of the programs in Phase III of the EST2Secretome. Gene Ontology (GO) [36] terms were assigned using BLAST2GO (v 1.6.2) [37]. Sequences were then mapped to biological pathways employing the KEGG Orthology-Based Annotation System (KOBAS) [38], with *C. elegans* data selected for the construction of background pathway maps. The query sequences were then compared using BLASTP against Wormpep v183 (e-value threshold of 1e-05). For each predicted ES sequence, the protein domain/family/motif was mapped using InterProScan [39], including 13 member databases, and the results were tabulated in decreasing order of abundance. Inferred ES protein sequence data were queried against the IntAct database (version 1.7.0) [32] to retrieve all interaction partners (e-value threshold of 1e-05). A comparison of homologues, based on BLAST scores from three different datasets, can be efficiently compared and presented visually using the program SimiTri [40]. In the case of parasitic nematodes, we generated BLAST-indexed datasets for the host organisms (human, other mammals or plant), *C. elegans* as the primary reference organism for nematodes and parasitic nematodes, based on NCBI protein datasets (defined by keyword), followed by local processing to add or remove selected organisms.

## Results/Discussion

### Identification of ES protein sequences from parasitic nematode ESTs

**Figure 2:**
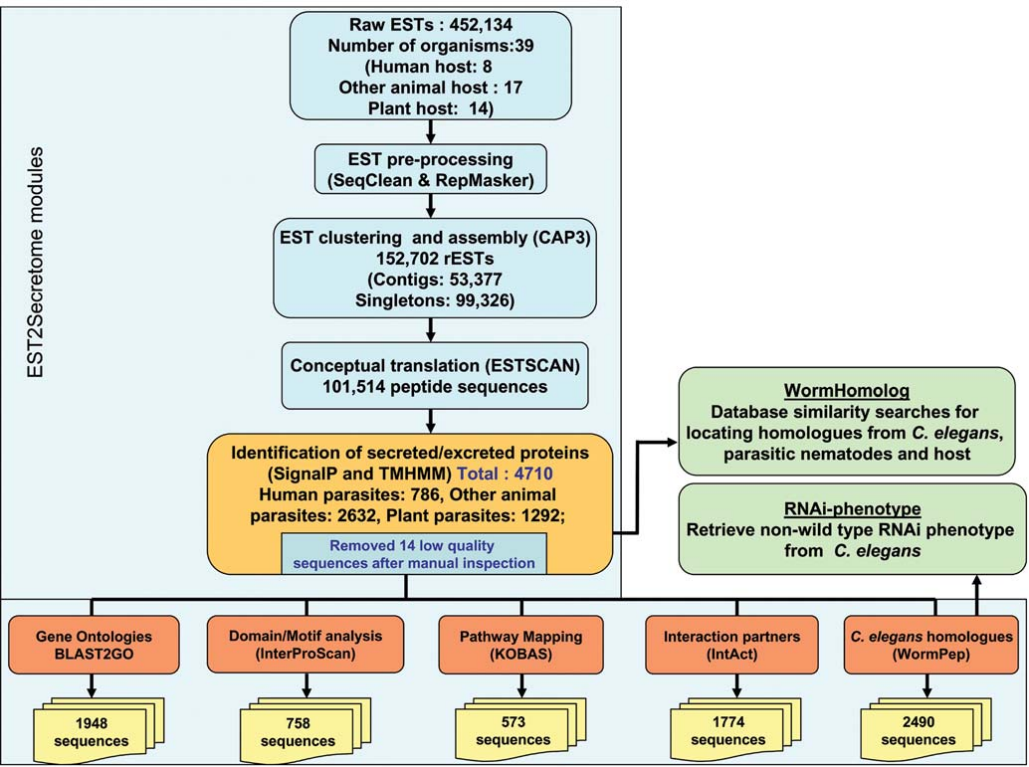
Identification and analysis of putative excretory-secretory proteins from parasitic nematode EST datasets. The “input” EST dataset and the results obtained from each step of the workflow are shown. All of these steps, excluding two nematode-specific steps (WormHomolog and RNAi-Phenotype), are currently incorporated within EST2Secretome.

EST2Secretome made possible the large-scale analysis and annotation of all publicly available EST data for nematodes that are parasitic in humans, other animals and plants. In total, 452,134 ESTs from 39 parasitic nematodes were downloaded from dbEST [19]. The organisms were broadly categorised on the basis of the host(s) they infect (Table 1) with seven, 18 and 14 nematodes parasitic in humans, other animals and plants, respectively, being selected for secretome analysis. Putative ES proteins were identified in the first two phases of EST2Secretome (see Figure 2). Phase I pre-processing and assembly resulted in a total of 152,702 representative ESTs (rESTs) comprising 53,377 contigs and 99,326 singletons, with 152,702 rESTs being conceptually translated into 101,514 peptide sequences. In Phase II, these conceptually translated peptide sequences were first analysed for the presence of N-terminal signal peptide, followed by the absence of transmembrane helices. We thus identified a total of 4,710 putative soluble ES proteins (2,632 in animal-, 1,292 in plant- and 786 in human-parasitic nematodes) (see Table 2), representing 4.6% of the total number of putative sequences identified. This result is comparable with earlier single organism studies of the bovine lungworm, *D. viviparus* [23], in which 85 secreted proteins were identified (representing 5.0% of 1685 peptides) and *T. vitirinus* [24], in which 40 secreted proteins were identified (representing 6.2% of 640 proteins). We manually examined the ES protein sequence data and found that 14 of 4710 entries were low quality sequences containing predominantly long stretches of unknown amino acids (X's), as a result of repeat masking, followed by conceptual translation. These sequences were from organisms like *Meloidogyne chitwoodi* and *Pratylenchus vulnus* which lack repeat libraries. These 14 sequences were functionally analysed and annotated in the EST2Secretome pipeline but could not be assigned any function. This step represents one of the challenges involved in the computational analysis of single pass reads from any organism which is not well characterized based on genomic data.

**Table 2:** Summary of EST2Secretome's Phase I and Phase II results for human, other animal and plant nematode parasites.

Number	Parasitic nematode	ESTs	Contigs	Singletons	rESTs	Putative peptides	Putative ES proteins	Percentage of ES proteins /species
**Human parasites**								
1	*Ancylostoma ceylanicum*	10712	1241	2111	3351	2873	212	7.3
2	*Ascaris lumbricoides*	1863	247	565	812	379	18	4.7
3	*Brugia malayi*	26215	2309	8108	10417	4240	149	3.5
4	*Necator americanus*	5032	728	1650	2378	1533	118	7.7
5	*Onchocerca volvulus*	14974	1143	4804	5947	2863	188	6.5
6	*Strongyloides stercoralis*	11392	1510	2743	4253	3434	61	1.7
7	*Wuchereria bancrofti*	4847	395	1562	1957	1152	39	3.3
**Parasites of other animals**								
1	*Ancylostoma caninum*	80551	10823	11960	22783	15731	510	3.2
2	*Ascaris suum*	40771	2936	5455	8391	5314	366	6.8
3	*Dirofilaria immitis*	4005	521	1166	1687	999	22	2.2
4	*Dictyocaulus viviparus*	4469	475	2194	2669	1550	80	5.1
5	*Haemonchus contortus*	21975	1910	2422	4332	3589	344	9.5
6	*Litomosoides sigmodontis*	2699	372	1123	1495	1188	54	4.5
7	*Nippostrongylus brasiliensis*	8238	969	1960	2929	1898	197	10.3
8	*Ostertagia ostertagi*	7006	786	1465	2251	1735	110	6.3
9	*Oesophagostomum dentatum*	328	133	314	447	374	44	11.7
10	*Onchocerca ochengi*	60	4	48	52	26	1	3.8
11	*Parastrongyloides trichosuri*	7963	941	2280	3221	2346	98	4.1
12	*Strongyloides ratti*	14761	1392	3353	4745	2716	74	2.7
13	*Teladorsagia circumcincta*	6058	736	1392	2128	1733	259	14.9
14	*Toxocara canis*	4889	617	854	1471	994	74	7.4
15	*Trichinella spiralis*	25268	3274	3894	7168	5328	211	3.9
16	*Trichuris muris*	2714	436	1067	1503	1262	87	6.8
17	*Trichuris vulpis*	3063	363	953	1316	944	76	8.0
18	*Trichostrongylus vitrinus*	1000	324	388	712	555	26	4.6
**Plant parasites**								
1	*Globodera pallida*	*4378*	*482*	*2585*	*3067*	*2064*	*92*	4.4
2	*Globodera rostochiensis*	*11851*	*2008*	*2258*	*4266*	*3301*	*121*	3.6
3	*Heterodera glycines*	*24444*	*3735*	*4800*	*8535*	*7019*	*260*	3.7
4	*Heterodera schachtii*	*2818*	*449*	*821*	*1270*	*1014*	*40*	3.9
5	*Meloidogyne arenaria*	*5018*	*655*	*1944*	*2599*	*1635*	*39*	2.3
6	*Meloidogyne chitwoodi*	*12218*	*1275*	*3787*	*5062*	*2339*	*65*	2.7
7	*Meloidogyne hapla*	*24452*	*2938*	*3936*	*6874*	*4153*	*143*	3.4
8	*Meloidogyne incognita*	*20334*	*2462*	*3236*	*5698*	*3940*	*111*	2.8
9	*Meloidogyne javanica*	*7587*	*946*	*2558*	*3504*	*1836*	*61*	3.3
10	*Meloidogyne paranaensis*	*3710*	*722*	*936*	*1658*	*1085*	*27*	2.4
11	*Pratylenchus penetrans*	*1928*	*251*	*186*	*437*	*406*	*26*	6.4
12	*Pratylenchus vulnus*	*5812*	*490*	*2243*	*2733*	*1232*	*47*	3.8
13	*Radopholus similis*	*7380*	*1152*	*2896*	*4048*	*2809*	*75*	2.6
14	*Xiphinema index*	*9351*	*1227*	*3309*	*4536*	*3925*	*185*	4.7

Details of the EST data obtained from dbEST, the contigs and singletons generated by preprocessing, overall representative ESTs (rESTs), peptides from conceptual translation and putative excretory-secretory (ES) proteins identified are provided.

We employed EST2Secretome for the analysis of the entire proteome (23,624 sequences) of the model free living nematode, *C. elegans*, in the Wormpep database (18^th^ February 2008). 2,649 (11.2%) sequences were predicted to be ES proteins, which is in the range of 8–20% suggested by Grimmond et al. [16]. These results independently validated the ability of the EST2Secretome pipeline to correctly identify ES proteins, using the Phase II filtering steps. The lower percentage of 4.6% ES proteins from EST data compared to 11.2% in *C. elegans* could be due the low coverage of the entire protein-coding gene set, compared to entire proteome comprising full length protein sequences in *C. elegans*, or to the low quality of some ESTs in public databases.

### Analysis of putative excretory-secretory proteins

We carried out a comprehensive analysis of the 4,710 ES proteins predicted, using all relevant components of Phase III in EST2Secretome as well as some additional bioinformatic tools specific to nematodes (Figure 2). Functional annotation comprised the assignment of GO terms and pathway associations using KEGG pathways; mapping protein domains/motifs, with a particular focus on nematode-specificity and identifying protein interaction partners. Subsequently, we used comparative genomics approaches to identify orthologues in the free-living nematode *C. elegans*, with their associated loss-of-function RNAi phenotypes. From database comparisons with human, other animal and plant host sequences, we predicted several ES proteins that were either absent from their host or distantly related to host homologues, which might represent potential novel drug or vaccine targets for parasite intervention. Results of these analyses are described in the following sections.

### Functional classification of excretory-secretory proteins

#### Gene Ontology (GO)

GO has been used widely to predict gene function and classification. It provides a dynamic vocabulary and hierarchy that unifies descriptions of biological, cellular and molecular functions across genomes. BLAST2GO [37], is a sequence-based tool to assign GO terms, extracting them for each BLAST-match obtained by mapping to extant annotation associations. Using the BLAST2GO module of EST2Secretome, we could functionally assign GO terms to 1,948 (41%) of 4,710 putative ES proteins. The efficacy of GO annotations reported here is comparable to 43% obtained for ES proteins from 80,551 *A. caninum* ESTs. A total of 551 ES and 15,221 non-ES proteins were defined, to which our pipeline could assign function GO terms to 43% and 51%, respectively. The difference in the extent of functional annotation could be attributed to many uncharacterized (appear to be novel) proteins in ES proteins compared to non-ES proteins.

**Figure 3:**
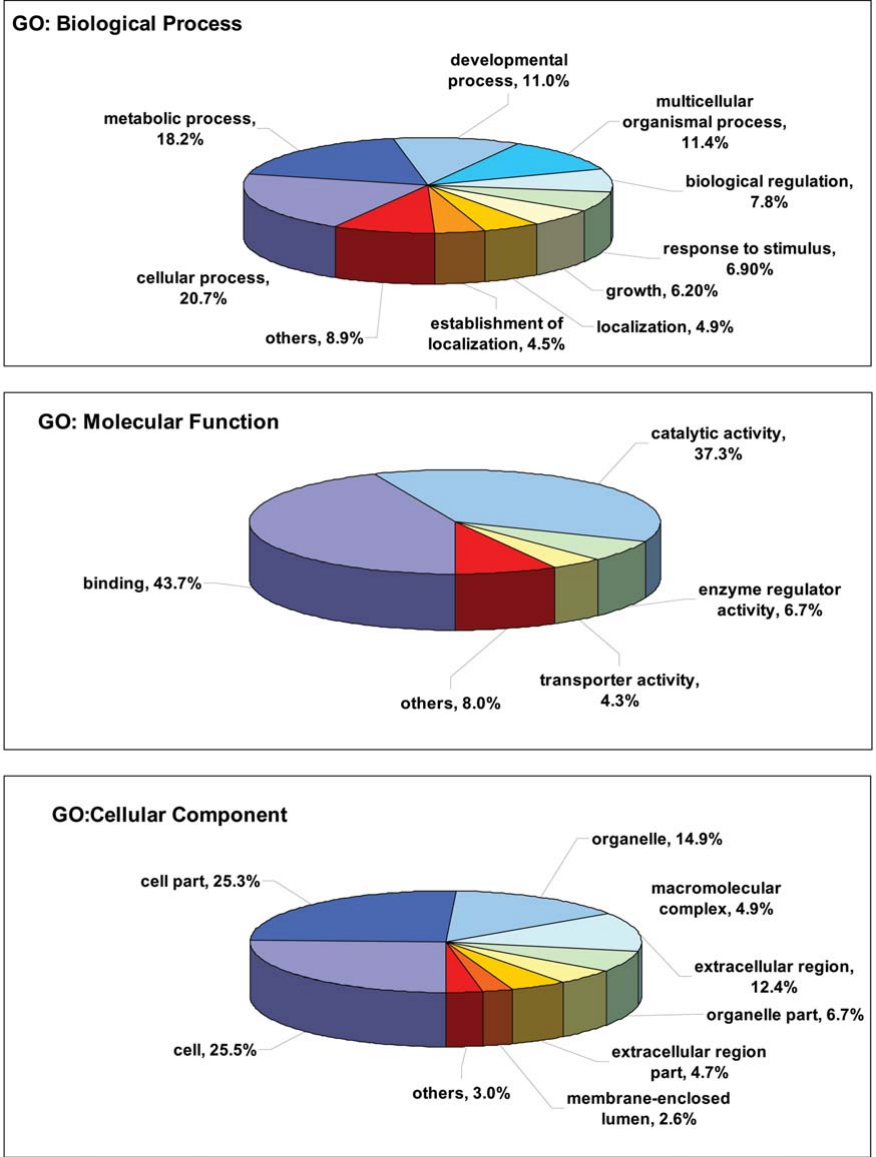
Assignment of Gene Ontology (GO) terms for putative excretory-secretory proteins. Components, such as Biological Process, Molecular Function and Cellular Component, are indicated. Individual GO categories can have multiple mappings. Percentages shown reflect the total categories annotated and not the total sequences annotated under each component.

For our parasitic nematode dataset, the 1,948 ES sequences with GO annotations could be annotated further, with 1,092 being assigned biological process (BP), 1,210 molecular function (MF) and 779 cellular component (CC) GO terms. A summary of GO annotation by biological process, cellular component and molecular function is provided in Figure 3. When we examined the GO terms in detail, we found that more than half of the sequences (420/779) were annotated specifically with terms pertaining to the extracellular region (GO: 0005576), including extracellular matrix (GO: 0031012), extracellular matrix part (GO: 0044420), extracellular space (GO: 0005615) and extracellular region part (GO: 0044421). While each sequence was annotated with multiple cellular component terms, leading to 18% overall instances of “extracellular” among the total 2285 cellular component terms, these annotations strengthened the computational prediction of ES proteins from EST datasets. We also validated the GO terms for overall instances of the GO term “extracellular” by comparing with 2,649 inferred ES proteins derived from *C. elegans* proteome. We assigned GO terms to these ES proteins and found an overall percentage of 29% of “extracellular” GO terms in the *C. elegans* proteome (data not shown). The higher percentage in *C. elegans* dataset could be due to the use of full-length protein sequences from *C. elegans*, compared with the dataset analysed, which is derived exclusively from ESTs. Amongst the most common GO categories representing biological processes were metabolic process (GO: 0008152) and cellular process (GO: 0009987). The largest number of GO terms in molecular function was binding (GO: 0005488) and catalytic activity (GO: 0003824), both of which are significant from the viewpoint of identifying novel drug or vaccine candidates. A complete listing of GO mappings assigned to ES protein data is provided in Table S1.

#### Pathway mapping

Biochemical functionality can also be categorised by assigning sequences to biological pathways using the Kyoto Encyclopedia of Genes and Genomes database (KEGG) [41]. We utilised KEGG orthology (KO) terms and predicted putative functionality by mapping putative ES proteins to KEGG pathways, using the KOBAS [38] module in EST2Secretome, with an e-value cut-off of 1.0e-5 (default). A total of 573 (12.2%) sequences were mapped to 138 KEGG pathways. The top 25 ‘highly represented’ pathways, ranked according to number of putative ES proteins mapped, are shown in Table 3. Molecular entities linked to protein folding and associated processing (n = 43) or antigen processing and presentation (n = 41) had the highest representation for the sequences mapped to KEGG pathways. Some of the other pathways that were well represented by ES proteins included the ribosome pathway (n = 17), the MAPK signalling pathway (n = 13), glutathione metabolism (n = 12), starch and sucrose metabolism (n = 12) and purine metabolism (n = 10). In the range of 1–5 entries, ES proteins were mapped to several pathways, including signal transduction mechanisms; GnRH signaling pathway; linoleic acid metabolism; N-glycan biosynthesis; ATP synthesis; aminosugar metabolism; galactose metabolism; glycine, serine and threonine metabolism. Even though not well represented, their identification as potential players in biological pathways could improve our understanding of nematode biology and assist in identifying essential proteins required in each pathway. Proteins (n = 41) predicted to be involved in antigen processing and presentation proteins or complement and coagulation cascades (n = 6) might play critical roles in host-parasite interactions. Although at this point, the precise roles of such molecules in the parasite-host interplay are unclear, some of them could be involved in manipulating or evading the immune response(s) in the host or associated with the parasite's immune response, suggesting avenues for future experimental work. Furthermore, we identified families of proteins representing serine, cysteine and metallo-proteinases as well as proteinase inhibitors (also supported by domain analysis). These categories have been considered as important targets for parasite invention and control [42],[43],[44]. Their occurrence in available EST data sets suggests that they are candidates for *in vitro* and *in vivo* studies. While these enzymes are inferred to mediate or modulate proteolytic functions, which, in turn, may facilitate tissue migration and other interactions with host cells, the proteinase inhibitors might protect the parasite against digestion by endogenous or host-derived proteinases [45]. A complete listing of the KEGG mappings to all the pathways and corresponding ES proteins is available as supplementary data (Table S2).

**Table 3:** Top 25 selected metabolic pathways in excretory-secretory proteins mapped using KEGG database.

Number	KEGG Pathway	ES proteins
1	Protein folding and associated processing	43
2	Antigen processing and presentation	41
3	Arachidonic acid metabolism	17
4	Ribosome	17
5	MAPK signaling pathway	13
6	Glutathione metabolism	12
7	Starch and sucrose metabolism	12
8	Amyotrophic lateral sclerosis (ALS)	10
9	Purine metabolism	10
10	Other amino acid metabolism	9
11	N-Glycan biosynthesis	9
12	Oxidative phosphorylation	9
13	VEGF signaling pathway	8
14	Pyrimidine metabolism	8
15	GnRH signaling pathway	8
16	Linoleic acid metabolism	7
17	Complement and coagulation cascades	6
18	Pores ion channels	6
19	Ether lipid metabolism	6
20	Fc epsilon RI signaling pathway	6
21	Neuroactive ligand-receptor interaction	6
22	Glycerophospholipid metabolism	6
23	ATP synthesis	6
24	Calcium signaling pathway	6
25	Long-term depression	6

#### Analysis of protein domains and motifs using InterProScan

Assignment of protein function is strengthened by matching the query sequence to specific secondary databases containing information on protein domains/motifs/signatures, as this step adds value to the annotation by pin-pointing a domain/motif or region in a protein sequence characteristic for a particular protein family. In this study, we interrogated all 13 InterPro member databases [46] using the InterProScan [39] module of EST2Secretome, to map protein domain/motifs for the entire ES protein dataset. The top 20 representative protein families with species coverage are given in Table 4, and a full list of all of the protein families, domains, active sites is provided in Table S3. The “transthyretin-like” family of proteins was amongst the most represented, comprising 153 ES protein entries and being present in 31 species. This family has been classified as nematode-specific and is also called “family 2”, based on a pioneering genome-wide study of *C. elegans* by Sonnhammer and Durbin [47]. The inferred proteins showed a weak homology to transthyretin (formerly called pre-albumin) which transports thyroid hormones [47]. Another highly represented group of domains was the “chromadorea ALT family”, identified in 90 ES proteins in seven species (Table 4). This family consists of several ALT protein homologues, found specifically in nematodes [48]. Two well-known members of this family, ALT-1 and the closely related ALT-2, have been found to be candidates for a vaccine against human filariasis [48]. Some of the other well-represented domain families in the present datasets were papain peptidase C1A, protease inhibitor I35, peptidase A1 and galectin, which were not predicted to be parasite- or nematode-specific unlike the nematode fatty acid retinoid binding family. However, there is enormous redundancy in the InterProScan results, due to the overlap in the family, domain, pattern and motif definitions from the member databases. For example, we note that the allergen V5/Tpx-1 family contains the Ves allergen family (Table S3) and is also the “parent” (using InterPro nomenclature) of the highly represented SCP-like extracellular domain (131 sequences from 23 species) which form part of the superfamily of the pathogenesis-related proteins (PRPs) [49],[50]. Similarly, the papain peptidase C1A family (Table 4) contains the papain C-terminal domain of peptidase C1A as well as the cysteine peptidase active site (Table S3), while the globin-like family (14 members, Table 4) contains the globin family (11 members, Table S3). While studying such molecules could deepen our understanding of host–parasite relationships, the interdependencies between the various functional assignments afforded by InterProScan need to be unravelled to ascertain the exact significance of these functional domain definitions.

**Table 4:** Top 20 nonredundant protein families of known function found in excretory-secretory proteins.

Number	InterProScan ID	Description	Type	ES sequences	Species coverage
1	IPR001534	Transthyretin-like	Family	153	31
2	IPR001283	Allergen V5/Tpx-1 related	Family	111	21
3	IPR013128	Peptidase C1A, papain	Family	96	23
4	IPR008451	Chromadorea ALT	Family	90	7
5	IPR002544	FMRFamide-related peptide	Family	35	17
6	IPR001820	Proteinase inhibitor I35, tissue inhibitor of metalloproteinase	Family	25	10
7	IPR001461	Peptidase A1	Family	22	12
8	IPR008632	Nematode fatty acid retinoid binding	Family	18	13
9	IPR009050	Globin-like	Family	14	7
10	IPR004947	Deoxyribonuclease II	Family	14	5
11	IPR002198	Short-chain dehydrogenase/reductase SDR	Family	14	11
12	IPR001211	Phospholipase A2	Family	13	8
13	IPR000889	Glutathione peroxidase	Family	12	10
14	IPR001547	Glycoside hydrolase, family 5	Family	12	8
15	IPR000215	Proteinase inhibitor I4, serpin	Family	12	6
16	IPR008597	Destabilase	Family	11	10
17	IPR000480	Glutelin	Family	11	10
18	IPR001580	Calreticulin/calnexin	Family	11	10
19	IPR000720	Peptidyl-glycine alpha-amidating monooxygenase	Family	11	9
20	IPR001079	Galectin, galactose-binding lectin	Family	10	10

#### Identification of interaction partners: the parasite interactome

Although each protein sequence was annotated individually, it is important to study proteins as part of larger protein complexes and pathways within a cell. By studying each protein and its binding partners in the context of a network, insights into possible functions within a cell can be gleaned. Moreover, protein interactions provide a valuable resource for the elucidation of cellular function, and there is enormous interest in identifying protein interaction partners as a means of understanding the complexities of a cell. In the context of the current analysis, it is even more important to study protein–protein interactions, as a complex interplay exists between the cellular environments of the parasite and its host during the course of invasion and infection. Furthermore, the understanding of the host and parasite interactions at the protein level could identify novel “cross-talk” between previously unlinked pathways as well as facilitate the discovery of new drug targets. Molecular interactions of protein pairs in one organism are expected to be conserved in other related organisms and can be derived based on sequence-based searches for conserved protein–protein interactions or “interlogs” [51]. Interspecies comparative studies among human, yeast, free-living worm (*C. elegans*) and fly have conserved protein interactions and, in turn, conserved sub-networks [52],[53]. Using a similar approach, we initially obtained protein interaction data from the IntAct database [32], and queried the 4,710 protein sequences in this database using BLASTP (with an e-value threshold of 1e-05). From the ES dataset for parasitic nematodes, 1,774 (37.6%) sequences had homologues in IntAct data, with at least one interaction partner (Table S4). The most similar IntAct sequences (with an e-value ≤1e-100) and all of their corresponding interaction partners are listed in Table 5. In the present analysis, different levels of complexity were found in the patterns of interactions. Heat-shock proteins, cathepsins, ribosomal protein subunits and enzymes, such as glyceraldehyde-3-phosphate dehydrogenase 3, dolichyl glycosyltransferase, were highly connected through primary interaction partners and, in turn, to several secondary interaction partners. Interestingly, we found a small number of partially characterized, yet to be studied entries, such as *cpl*-1, *egl*-21, *ile*-1, *ccg*-1, *gln*-6, *cut*-3 and *pdi*-3 in the range of one to four interaction partners. Finally, we also found proteins commonly present in parasitic nematodes, such as calreticulins, calumenin-like proteins and aspartyl proteases that had 2 to 10 interaction partners. A graphic representation of the interaction network of cathepsin Z protein 1 and its primary and secondary interaction partners is shown in Figure S1. While these data are useful, each of these interactions needs to be investigated experimentally to understand the role of these molecules *in vivo*.

**Table 5:** Identification of interaction partners: selected entries identified during the comparison and their interaction partners obtained using IntAct database.

Sequence ID	E-value	Top homolog in IntAct database (ID)	Description	Number of interaction partners
Ancylostoma_caninum_Contig10288	1.00E-144	EBI-312868	uncharacterized protein	5
Ancylostoma_caninum_Contig4711	1.00E-133	EBI-320128	Calumenin-like protein	1
Ancylostoma_caninum_Contig7345	0	EBI-319290	fumarate hydratase	2
Ancylostoma_caninum_Contig9959	1.00E-154	EBI-315239	Cathepsin z protein 1	3
Ancylostoma_ceylanicum_Contig10	1.00E-112	EBI-317252	uncharacterized protein ccg-1	1
Ancylostoma_ceylanicum_Contig113	1.00E-123	EBI-315917	Heat shock 70 kDa protein C precursor	2
Ancylostoma_ceylanicum_Contig153	0	EBI-319290	fumarate hydratase	2
Ancylostoma_ceylanicum_Contig249	0	EBI-323711	Protein disulfide-isomerase 2 precursor	2
Ancylostoma_ceylanicum_Contig364	1.00E-105	EBI-314435	Calreticulin precursor	7
Ancylostoma_ceylanicum_Contig669	1.00E-159	EBI-315958	uncharacterized protein cpl-1	8
Ancylostoma_ceylanicum_Contig713	0	EBI-318186	uncharacterized protein egl-21	2
Ascaris_suum_Contig2628	1.00E-138	EBI-314454	Fructose-bisphosphate aldolase 2	8
Brugia_malayi_Contig1642	1.00E-119	EBI-316122	60S ribosomal protein L3	10
Brugia_malayi_Contig2261	1.00E-114	EBI-314435	Calreticulin precursor	7
Dictyocaulus_viviparus_Contig403	1.00E-112	EBI-315261	Aspartyl protease protein 2, isoform a	4
Globodera_pallida_Contig133	1.00E-101	EBI-319315	N-acetylgalactosaminyltransferase 8	2
Haemonchus_contortus_Contig1874	1.00E-135	EBI-314435	Calreticulin precursor	7
Heterodera_glycines_Contig1053	1.00E-109	EBI-315958	uncharacterized protein cpl-1	8
Heterodera_glycines_Contig609	1.00E-104	EBI-358866	Dolichyl-diphosphooligosaccharide–protein glycosyltransferase 48 kDa subunit precursor	16
Litomosoides_sigmodontis_Contig142	1.00E-136	EBI-318186	uncharacterized protein egl-21	2
Meloidogyne_arenaria_Contig265	1.00E-139	EBI-330517	uncharacterized protein cut-3	1
Meloidogyne_hapla_Contig1559	1.00E-131	EBI-198835	Vacuolar ATP synthase subunit B	4
Meloidogyne_incognita_Contig2289	1.00E-122	EBI-322730	uncharacterized protein	1
Meloidogyne_paranaensis_Contig210	1.00E-130	EBI-330517	uncharacterized protein cut-3	1
Necator_americanus_Contig405	0	EBI-318186	uncharacterized protein egl-21	2
Nippostrongylus_brasiliensis|EH361309	1.00E-121	EBI-320128	Calumenin-like protein	1
Onchocerca_volvulus_Contig549	1.00E-110	EBI-314435	Calreticulin precursor	7
Ostertagia_ostertagi_Contig746	1.00E-123	EBI-313057	Temporarily assigned gene name protein 196	1
Strongyloides_ratti_Contig1052	1.00E-131	EBI-314435	Calreticulin precursor	7
Strongyloides_ratti_Contig356	1.00E-135	EBI-323711	Protein disulfide-isomerase 2 precursor	2
Strongyloides_stercoralis_Contig19	1.00E-151	EBI-322448	Heat shock 70 kDa protein A	5
Teladorsagia_circumcincta_Contig366	1.00E-102	EBI-323711	Protein disulfide-isomerase 2 precursor	2
Teladorsagia_circumcincta_Contig443	1.00E-115	EBI-315239	Cathepsin z protein 1	3
Trichinella_spiralis_Contig698	1.00E-110	EBI-1049597	Calreticulin precursor	7
Trichuris_muris|BM174688	1.00E-111	EBI-352338	Serine/threonine-protein phosphatase PP1-beta catalytic subunit	6
Wuchereria_bancrofti_Contig268	1.00E-114	EBI-314435	Calreticulin precursor	7

### Comparison with the free-living nematode, *C. elegans*, and associated RNAi phenotype information

*C. elegans* represents the best characterized nematode in many respects, particularly in terms of its genome, genetics, biology, physiology and biochemistry [31],[54],[55]. In addition, *C. elegans* (non-wild-type or loss-of-function) RNAi phenotypes may provide indications of the relevance and function(s) of homologous genes in other nematodes (of animals) for which the complexity of an obligate parasitic life cycle and the lack of an effective *in vitro* culture system and/or an RNAi assay make high-throughput screening impractical [56]. Moreover, the set of genes with RNAi loss-of-function phenotypes constitutes a pool of significant and potentially essential *C. elegans* genes. The RNAi phenotype data, comprising, ∼62,000 entries (on 10 January 2008), is available to download through WormBase [31]. In this study, we compared the 4,710 predicted ES proteins to the *C. elegans* proteome using BLASTP program and predicted 2,490 (52.8%) homologues in *C. elegans* (threshold e-value of 1e-05). From these 2,490 *C. elegans* homologues, we retrieved exclusively protein entries that had been reported with any one of the following observed strong RNAi phenotypes: Emb (embryonic lethal, including pleiotropic defects severe early emb), Lvl (larval lethal), Lva (larval arrest), Stp (sterile progeny), Ste (maternal sterile) and Gro (slow growth). In the present dataset (available from Table S5), 267 *C. elegans* homologues were identified that had one or more observed “strong” loss-of-function phenotype in RNAi; selected examples are listed in Table 6. The latter RNAi phenotypes were selected as they have been inferred to be essential for nematode survival or growth [56],[57], also representing potential drug and/or vaccine targets.

**Table 6:** Selected ES proteins with non-wild-type *C. elegans* RNAi phenotype.

Sequence ID	Description and SwissProt ID	E-Value	% Identity	WBGene ID	RNAi phenotype in *C. elegans*
Ancylostoma ceylanicum Contig113	locus:hsp-3 heat shock protein status: SW:P27420	1.00E-123	90	WBGene00002007	EMB embryonic lethal, GRO slow growth, LVA larval arrest, EMB embryo osmotic integrity abnormal early emb
Brugia malayi Contig2071	N-oligosaccharyl transferase 48kd subunit status: SW:P45971	3.00E-46	48	WBGene00011638	GRO slow growth, EMB embryonic lethal, LVL larval lethal, EMB embryo osmotic integrity abnormal early emb
Necator americanus Contig572	locus:ppn-1 SW:O76840 protein_id:AAM29666.1	1.00E-09	41	WBGene00016498	GRO slow growth, EMB embryonic lethal, STP sterile progeny, LVA larval arrest, LVL larval lethal
Onchocerca volvulus Contig306	locus:dnj-12 DnaJ, prokaryotic heat shock protein TR:O45502	7.00E-18	60	WBGene00001030	EMB embryonic lethal, EMB mitotic spindle abnormal early emb, EMB embryonic lethal
Strongyloides stercoralis Contig19	locus:hsp-1 HSP-1 heat shock 70kd protein A status: SW:P09446	1.00E-152	89	WBGene00002005	EMB embryonic lethal, LVA larval arrest
Wuchereria bancrofti Contig317	locus:eif-3.G RNA binding protein status: SW:Q19706	6.00E-34	41	WBGene00001230	GRO slow growth, EMB embryonic lethal, STP sterile progeny, LVA larval arrest, EMB pleiotropic defects severe early emb
Ascaris suum Contig194	locus:arx-3 TR:Q9U1R7	3.00E-75	68	WBGene00000201	EMB embryonic lethal, GRO slow growth LVL larval lethal
Dictyocaulus viviparus Contig352	locus:pyp-1 inorganic pyrophosphatase SW:Q18680	1.00E-102	70	WBGene00008149	GRO slow growth, EMB embryonic lethal, EMB embryonic lethal, LVA larval arrest, LVL larval lethal
Haemonchus contortus Contig942	locus:rps-22 40S ribosomal protein status: TR:O17218	3.00E-62	88	WBGene00004491	EMB embryonic lethal, GRO slow growth, STP sterile progeny, LVA larval arrest, EMB pleiotropic defects severe early emb
Nippostrongylus brasiliensis Contig922	locus:pas-3 endopeptidase status:Confirmed SW:Q9N599	1.00E-110	86	WBGene00003924	GRO slow growth, EMB embryonic lethal, LVA larval arrest, EMB passage through meiosis abnormal early emb
Ostertagia ostertagi L3|BQ625812	locus:flp-2 status: TR:O61465	2.00E-08	64	WBGene00001445	EMB embryonic lethal, LVA larval arrest, GRO slow growth, EMB embryonic lethal
Parastrongyloides trichosuri FL|BM513234	phosphate carrier protein precursor status: SW:P40614	3.00E-94	79	WBGene00008505	EMB embryonic lethal, LVA larval arrest, GRO slow growth
Strongyloides ratti L2|BI323377	locus:wts-1 Protein kinase C terminal domain TR:O45797	2.00E-35	48	WBGene00007047	EMB embryonic lethal, GRO slow growth
Teladorsagia circumcincta Contig658	NADH-ubiquinone dehydrogenase 24 KD subunit status: SW:Q20719	8.00E-80	65	WBGene00009992	EMB embryonic lethal, LVA larval arrest
Toxocara canis Contig163	locus:lpd-9 TR:Q22641	3.00E-23	42	WBGene00003065	EMB embryonic lethal, LVL larval lethal, LVA larval arrest, GRO slow growth
Trichinella spiralis Contig231	glycotransferase TR:Q9GZH4	9.00E-48	40	WBGene00020683	GRO slow growth, EMB embryonic lethal, LVA larval arrest, EMB embryo osmotic integrity abnormal early emb
Trichuris muris cDNA|BM277526	locus:rps-15 40S ribosomal protein S15 SW:Q9XVP0	4.00E-48	61	WBGene00004484	GRO slow growth, EMB embryonic lethal, LVL larval lethal, LVA larval arrest, EMB pleiotropic defects severe early emb
Globodera rostochiensis Contig2005	Ribosomal protein L3 SW:P49404	1.00E-18	44	WBGene00016142	LVA larval arrest, GRO slow growth, EMB embryonic lethal, LVL early larval lethal
Heterodera glycines Contig921	locus:let-767 SW:Q09517	5.00E-80	48	WBGene00002891	LVA larval arrest, GRO slow growth, EMB embryonic lethal, LVA larval arrest, LVL early larval lethal
Meloidogyne incognita Contig1102	Signal peptidase SW:P34525	3.00E-41	45	WBGene00019679	EMB embryonic lethal, GRO slow growth, LVL larval lethal, EMB embryo osmotic integrity abnormal early emb
Pratylenchus vulnus NEM3|CV199205	locus:ran-4 nuclear transport factor 2 like SW:Q21735	3.00E-19	54	WBGene00004305	GRO slow growth, EMB embryonic lethal, LVA larval arrest, EMB pronuclear nuclear appearance abnormal early emb
Xiphinema index Contig1097	locus:cmd-1 calmodulin SW:O16305	6.00E-44	60	WBGene00000552	GRO slow growth, STP sterile progeny, EMB embryonic lethal

### Comparative analyses of ES proteins

**Figure 4:**
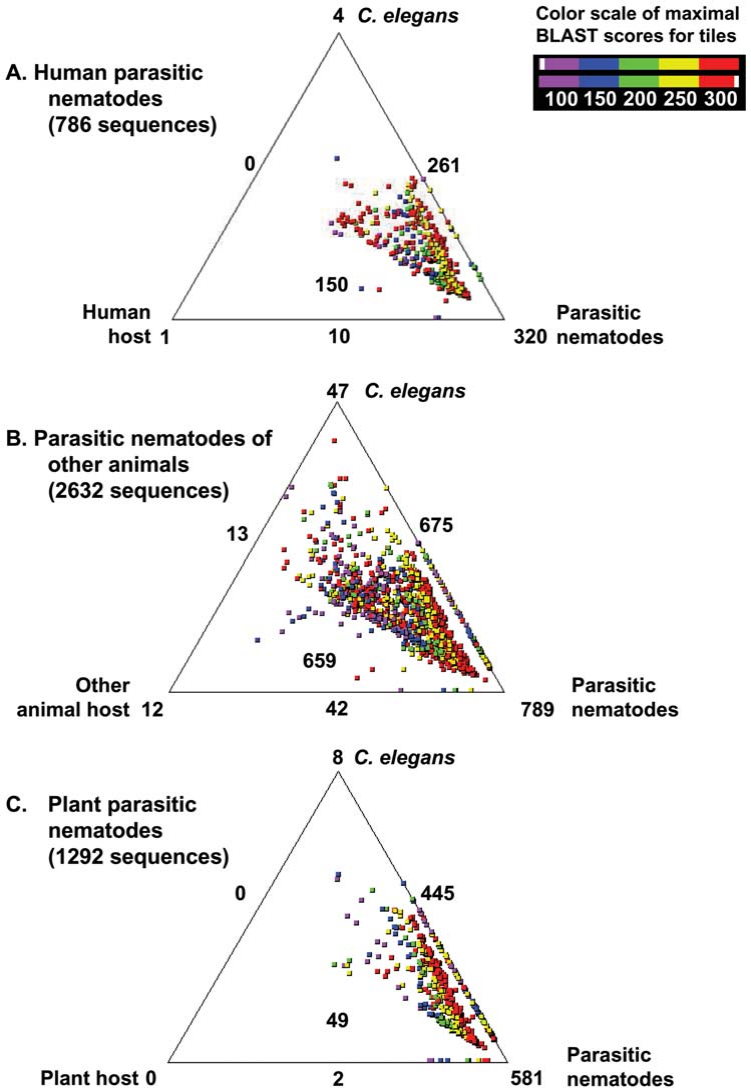
Comparison of ES proteins with the respective *C. elegans*, parasitic nematodes and host orthologues using SimiTri. Data for parasitic nematodes of A. humans, B. other animals or C. plants are presented, compared with their respective host organism. The numbers at each vertex indicate the number of ES proteins matching only the specific database. The numbers on the edges indicate the number of ES proteins matching the two databases linked by that edge. The boxed number within each triangle indicates the number of ES proteins with matches to all three datasets compared: *C. elegans*, parasitic nematodes and host databases.

Sequence-based searches were performed to classify the ES proteins, to identify the presence or absence of putative homologues in *C. elegans*, and to infer nematode-specific and parasite-specific genes. For parasitic nematodes, Parkinson et al. [40],[58] suggested previously that it is beneficial to make simultaneous three-way comparisons (using SimiTri) of a specific organism or a group of organisms with homologues in *C. elegans* (the ‘model nematode’), other nematode species as well as the host organism. Such an analysis provides a means for the rapid identification of genes/proteins conserved between any two datasets compared (e.g., between parasitic nematodes and free-living ones, or between parasitic nematode and its host). In the present study, we systematically compared inferred ES protein data with those available in three relevant databases. For the three ES protein datasets from nematodes parasitic in humans (786 proteins), animals (2,632 proteins) or plants (1,292 proteins), we selected *C. elegans* and parasitic nematode databases as well as databases specific to the host organisms for comparative analysis. For instance, data for parasitic nematodes of humans were matched with those of the human host, *C. elegans* and parasitic nematodes from other hosts. Similarly, ES proteins predicted for nematodes parasitic in animals or plants were compared against host datasets. Protein sequences available in the following three datasets (i) *C. elegans* (from Wormpep [31]), (ii) parasitic nematodes (constructed locally) and (iii) respective hosts (human, other animal and plants sequences from NCBI non-redundant protein database) were processed. Three-way comparison of the parasitic nematode database with homologues in *C. elegans*, their principal definitive host organism (human, other animal or plant) and the database of all available parasitic nematodes, have been presented using SimiTri [40] in Figure 4. In all three datasets for parasitic nematodes, inferred ES proteins congregated with parasitic nematodes rather than with *C. elegans* or with the host species (lower right hand corner of each triangle, coloured in red in Figure 4). Overall, 320 (40.7%), 789 (29.7%) and 581 (44.9%) ES proteins inferred from human-, other animal- and plant-parasitic nematodes were associated exclusively with parasitic nematodes and are interpreted to be parasite-specific, based on the data currently available. Of the homologues predicted to be nematode-specific (along the side of the triangle connecting *C. elegans* and parasitic nematodes), 585 (74.4%), 1,511 (57.4%) and 1,034 (80.0%) of the inferred ES proteins were confined to nematodes (based on currently available datasets). Based on these comparisons, we illustrate that a significant percentage of these proteins in parasitic nematodes are either parasite- or nematode-specific and are either absent from or very divergent in sequence from molecules in their host(s). These molecules might represent candidate targets for novel anthelmintics for parasite intervention. Importantly, their apparent specificity to parasitic nematodes or different groups within the phylum Nematoda renders them as important groups of molecules for future study, particularly in relation to the roles of these molecules in the host-parasite interplay, their involvement in inducing immune responses and disease in the host.

### Inferring potential drug/vaccine candidates from ES proteins

Based on evidence from the literature, we selected candidate molecules from parasitic nematodes which have already proven to be therapeutic or vaccine targets for scrutiny. Such targets are either in early phases of clinical trials or have been identified as candidates following detailed experimental study. Firstly, prominent anti-parasite vaccine candidates have been identified through the Human Hookworm Vaccine Initiative and include a family of pathogenesis-related (PR) proteins, such as the *Ancylostoma*-secreted proteins (ASPs) [59]. This initiative has characterized *Na*-ASP-2, a PR-1 protein, from *Necator americanus* [59] which is in Phase II clinical trials [60] and *Ac*-ASP-1 from *Ancylostoma caninum* which exhibits 97% identity to *Na*-ASP-2 [61]. Secondly, cathepsin L and Z-like cysteine proteases (known to have been implicated in moulting and tissue remodelling in free-living and parasitic nematodes) represent potential targets for onchocerciasis and have been studied in significant detail in *Onchocerca volvulus* [62],[63],[64]. Also, astacin-like metalloproteases (MTP) was selected, as L3s of parasitic nematodes secrete MTPs that are considered critical to invasion and establishment of the parasite in the host [65],[66]. Astacin-like MTPs, such as MTP-1, have been characterized mainly in *Ancylostoma caninum* and are secreted by infective hookworm larvae [66],[67]. The sequences for four such proteins were retrieved from NCBI and matched to the present ES dataset using BLASTP. We discovered likely homologues for all of these proteins in parasitic nematodes of humans, other animals and plants (Table 7); organisms for which there is published information on these proteins are indicated (in bold font). Based on the present analysis, we identified 12 homologues of *Ancylostoma*-secreted proteins (ASPs) (above the threshold e-value of 1e-05) in the datasets in following nematodes (Strongylida): *Necator americanus, Ancylostoma duodenale*, *Ancylostoma caninum, Haemonchus contortus* and *Teladorsagia circumcincta.* Of these, published reports are available for only *Necator americanus, Ancylostoma caninum, Haemonchus contortus* and *Ostertagia ostertagi* [7],[61],[65],[66], while the analysis, based exclusively on available data, showed that this group of proteins (inferred from ESTs) occurs in the parasitic nematodes *Teladorsagia circumcincta* and *Meloidogyne chitwoodi*. Moreover, we identified eleven cathepsin L-like cysteine proteases, nine cathepsin Z-like cysteine proteinases and eight astacin-like metalloproteases in ES protein datasets, providing novel, yet unpublished evidence for the presence of these proteins in a number of key parasitic nematodes of socio-economic importance.

**Table 7:** Example excretory-secretory proteins selected as potential drug/vaccine candidates based on literature evidence.

Molecules	Number of excretory-secretory proteins	Organisms represented
secreted protein	12	***Ancylostoma caninum***
ASP-2		***Haemonchus contortus,***
		*Meloidogyne chitwoodi,*
		***Necator americanus***
		***Ostertagia ostertagi***
		*Teladorsagia circumcincta*
cathepsin L-like cysteine protease	11	***Ancylostoma ceylanicum***
		***Ascaris suu***
		***Brugia malayi***
		***Dictyocaulus viviparus***
		***Heterodera glycines***
		*Meloidogyne javanica*
		*Ostertagia ostertagi*
		*Strongyloides ratti*
		*Teladorsagia circumcincta*
		*Trichuris muris*
		*Wuchereria bancrofti*
cathepsin Z-like cysteine proteinase	9	***Ancylostoma caninum***
		***Haemonchus contortus***
		*Parastrongyloides trichosuri*
		*Teladorsagia circumcincta*
		*Trichuris muris*
		*Xiphinema index*
astacin-like metalloprotease	8	***Ancylostoma caninum***
		***Ancylostoma ceylanicum***
		*Necator americanus*
		***Ostertagia ostertagi***
		***Strongyloides stercoralis***
		***Trichinella spiralis***

The table shows their occurrences in different nematode parasites inferred from ES protein analysis. Organisms with published evidence of these genes/proteins are shown in bold.

### Conclusion

In this study, based on a comprehensive, targeted analysis of almost 0.5 million publicly available ESTs, we have inferred and functionally annotated 4,710 putative ES proteins from 39 parasitic nematodes infecting humans, other animals or plants, using the EST2Secretome, a new workflow developed for the large-scale processing of EST and complete proteome data. Furthermore, EST2Secretome has been developed as a multi-purpose, high-throughput analysis pipeline for diverse applications. For instance, it is possible to conduct analyses of all predicted proteins containing only signal sequences by selecting only SignalP and deselecting the TMHMM option, or select only the TMHMM program to investigate transmembrane proteins. The option to enter protein sequence data alone into the pipeline is also useful following the direct sequencing of proteins in proteomic studies.

Detailed annotations of inferred ES proteins revealed several parasite-specific (being absent from *C. elegans* and the host) and nematode-specific molecules as potential drug or vaccine candidates. Included in this set of molecules are pathogen-related protein (PRP) domains and several novel, nematode-specific protein domains. Gene Ontology (GO) annotations, at the level of molecular function, revealed an overwhelming representation of binding (63.4%) and catalytic activity (54.1%), supporting the further biochemical, proteomic and/or functional characterization of the ES proteins inferred herein. Predicted protein interaction data for each ES protein enables the classification of molecules as essential for parasite existence or survival, with relative potential to serve as target for parasite intervention, based on the number of primary and secondary interaction partners, as well as those interactions that are specific to parasites, rendering such “hub proteins” as potential targets for functional studies. In order to predict which ES proteins are essential, we also categorised molecules according to “strong” loss-of-function RNA_i_ phenotypes for corresponding homologues in *C. elegans*. ES proteins homologous to these “loss-of-function” phenotypes are considered the best candidates for functional characterization, and possibly linked to the survival of the parasites. Finally, we selected some proteins for further characterization based on their similarity to proteins currently under evaluation as vaccines or drug targets. The present, systematic approach of inferring ES protein data from EST data sets represents a starting point for understanding the role ES proteins in parasitic nematodes and serves as a useful tool for the future study of essentially any eukaryotic organism.

## Supporting Information

Table S1 Assignment of Gene Ontology (GO) terms for putative excretory-secretory (ES) proteins, categorized according to Biological Process, Molecular Function and Cellular Component. Note that individual GO categories can have multiple mappings.
(0.04 MB XLS)

Table S2 Metabolic pathways in excretory-secretory proteins, mapped to Kyoto Encyclopedia of Genes and Genomes (KEGG) data.
(0.56 MB XLS)

Table S3 Representative protein domains/families found in excretory-secretory proteins identified using InterProScan.
(0.11 MB XLS)

Table S4 Identification of interaction partners: List of putative ES protein above the E-value threshold using top homologues from IntAct database.
(0.32 MB XLS)

Table S5 Comparison of excretory-secretory proteins with *C. elegans* proteome and identification of non-wild-type RNAi phenotypes obtained using WormBase.
(0.52 MB XLS)

Figure S1 Primary and secondary interaction partners for example ES protein, Cathepsin z protein 1: 137 molecules, 140 interactions.
(0.37 MB TIF)
