# Supplementary figures and images for: Needles in the EST Haystack: Large-Scale Identification and Analysis of Excretory-Secretory (ES) Proteins in Parasitic Nematodes Using Expressed Sequence Tags (ESTs)
Source: PLoS Negl Trop Dis. 2008 Sep 24;2(9):e301. doi: 10.1371/journal.pntd.0000301 (PMC2553489; doi:10.1371/journal.pntd.0000301)

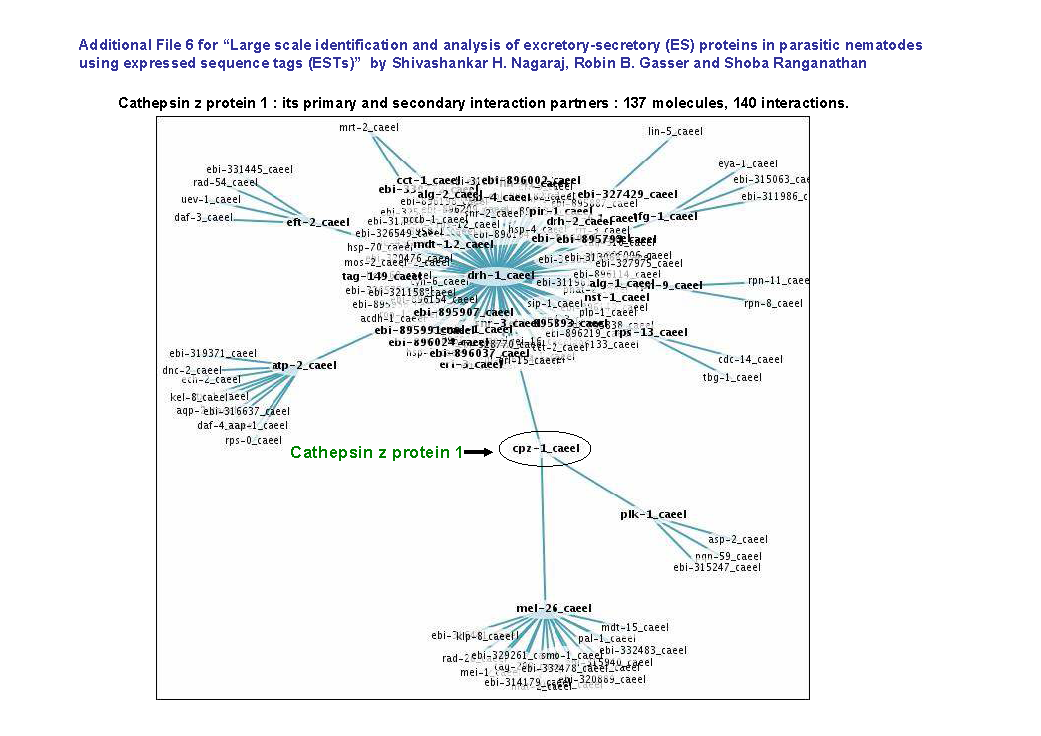

Supplement: Figure S1 — Primary and secondary interaction partners for example ES protein, Cathepsin z protein 1: 137 molecules, 140 interactions. (0.37 MB TIF) [file pntd.0000301.s006.tif]
